# Supplementary material for: Environmental versatility promotes modularity in genome-scale metabolic networks
Source: BMC Syst Biol. 2011 Aug 24;5:135. doi: 10.1186/1752-0509-5-135 (PMC3184077; doi:10.1186/1752-0509-5-135)
Supplement: Additional file 1 — Figures S1 to S7. This file contains the following additional figures: Figure S1 - MCMC sampling of genotypes with a given phenotype; Figure S2 - a FCS predominantly consists of a reactions belonging to one biochemical pathway; Figure S3 -Example of a frequently arising FCS in sampled genotypes; Figure S4 - Environmental versatility enhances the modularity index M regardless of the value of n; Figure S5 -Environmental versatility enhances the number of modules s regardless of the value of n; Figure S6 - Example of FCSs that account for the increase in modularity with versatility; Figure S7 - Schematic diagram describing nested sets of environments used to sample genotypes with decreasing Venv. [file 1752-0509-5-135-S1.DOC]

# Additional File 1: Figures S1 to S7

**Figure S1: The MCMC sampling of genotypes of a given phenotype. a)** A (discretized binary) metabolic network genotype refers to any subset of reactions taken from the global set of *N* reactions. Such a genotype can be represented as a bit string of length *N;* if the genotype has *n* reactions, the bit string will have *n* entries equal to 1 and all others equal to 0. **b)** For any such genotype, we can use *in silico* flux balance analysis (FBA) to determine the maximum possible biomass flux in a given environment. A genotype is considered *viable* in a given environment if and only if FBA predicts nonzero growth for that environment. A genotype has the desired phenotype if it is viable in a given list of environments. **c)** The set of all genotypes of given phenotype and having exactly *n* reactions forms a “genotype network”. The MCMC algorithm samples the genotype network via a long random walk among genotypes of interest, performing at each step a reaction swap. If the new genotype has the same phenotype, the step is accepted, otherwise the step is rejected. This process is repeated many times until an adequate sampling of the genotype network is obtained.

**Figure S2: A FCS predominantly consists of reactions belonging to one biochemical pathway.**  For each FCS obtained using the 1000 sampled genotypes with *V_env_* =89 and *n*=831 reactions (as in the *in silico* *E. coli* metabolic model), we determined the pathway annotation of each reaction it contained. If a FCS corresponds to a biochemical pathway, its reactions should predominantly have the same annotation. To test this hypothesis we defined the quantity *Q* for each FCS as the fraction of reactions sharing the dominant annotation for that FCS. Each FCS thus has a value of *Q*. From these values we can compute the cumulative distribution of *Q*, that is, the probability that *Q* is at least as large as a given value X. The resulting curve is shown here, along with the bisecting line. The intersection of the two shows that over 79% of the FCSs have a *Q* value of at least 0.79. A randomization test of the hypothesis that reaction annotations are not correlated within the FCSs rejects this hypothesis at P<0.001 (see Methods for details).

**Figure S3: Example of a frequently arising FCS in sampled genotypes; this FCS corresponds to a biochemical pathway.** We display here the most frequent FCS having more than 5 reactions. It occurs in 898 of the 1000 sampled genotypes with phenotype *V_env_* =89. All of its reactions belong to the histidine metabolism pathway. In this figure, the (hyper) edges represent reactions taking substrate metabolites to product metabolites (all these reactions are in fact irreversible). To reduce clutter, the ubiquitous high degree metabolites such as ATP, NADH, etc. have been omitted from this figure. Abbreviations of metabolite names are as follows: aicar = 5-Amino-1-(5-Phospho-D-ribosyl)imidazole-4-carboxamide; akg = 2-Oxoglutarate; eig3p = D-erythro-1-(Imidazol-4-yl)glycerol 3-phosphate; gln-L = L-Glutamine; glu-L = L-Glutamate; his-L = L-Histidine; hisp = L-Histidinol phosphate; histd = L-Histidinol; imacp = 3-(Imidazol-4-yl)-2-oxopropyl phosphate; prbamp = 1-(5-Phosphoribosyl)-AMP; prbatp = 1-(5-Phosphoribosyl)-ATP; prfp = 1-(5-Phosphoribosyl)-5-[(5-phosphoribosylamino)methylideneamino]imidazole-4-carboxamide; prlp = 5-[(5-phospho-1-deoxyribulos-1-ylamino)methylideneamino]-1-(5-phosphoribosyl)imidazole-4-carboxamide; prpp = 5-Phospho-alpha-D-ribose 1-diphosphate.

**Figure S4: Environmental versatility enhances the modularity index *M* regardless of the value of *n*.** Similar to what is shown in Figure 2a, we display here the trend for the mean of the modularity index *M* as a function of increasing *V_env_*, *i.e.*, as one forces genotypes to grow on more and more environments. **a)** Networks containing *n*=500 reactions. **b)** Networks containing *n*=700 reactions. We find the same qualitative increase in the average of *M* as *V_env_* increases, irrespective of the value of *n*.

**Figure S5: Environmental versatility enhances the number of modules *s* regardless of the value of *n*.** Similar to what is shown in Figure 2b, we display here the trend for the mean of *s* (the number of FCSs) as a function of increasing *V_env_*, *i.e.*, as one forces genotypes to grow on more and more environments. **a)** *n*=500 reactions. **b)** *n*=700 reactions. We find the same increase in the average of *s* as *V_env_* increases, irrespective of the value of *n*.

**Figure S6: Example of FCSs that account for the increase in modularity with versatility.** The set R_89_\R_1_ is constructed from FCSs that are over-represented at high versatility. The set contains 24 reactions that are as close as possible to nutrients (have a scope distance of 1). Starting with all the FCSs in our 1000 sampled genotypes, we selected those that contained at least one of the 24 reactions. Shown here are the corresponding most frequent FCSs subject to the constraint of containing 4 or more reactions. In this figure, the (hyper) edges represent reactions involving metabolites. Green edges represent irreversible reactions and red edges represent reversible reactions. To reduce clutter, the ubiquitous high degree metabolites such as ATP, NADH, etc. have been omitted from this figure. Abbreviations of metabolite names are as follows: 3hcinnm = 3-hydroxycinnamic acid; 3hcinnm(e) = 3-hydroxycinnamic acid (Extracellular); dhap = Dihydroxyacetone phosphate; dhcinnm = 2,3-dihydroxicinnamic acid; fc1p = L-Fuculose 1-phosphate; fcl-L = L-fuculose; fuc-L = L-Fucose; fuc-L(e) = L-Fucose (Extracellular); fum = Fumarate; hkntd = 2-hydroxy-6-ketononatrienedioate; lald-L = L-Lactaldehyde; op4en = 2-Oxopent-4-enoate; rml = L-Rhamnulose; rml1p = L-Rhamnulose 1-phosphate; rmn = L-Rhamnose; rmn(e) = L-Rhamnose (Extracellular).

**Figure S7: Schematic diagram describing nested sets of environments used to sample genotypes with decreasing *V_env_*.** Because the computations carried out here are very demanding in computing time, we have used only one subset of 70 environments within *V_env_* =89 to sample an ensemble with *V_env_* =70. Similarly, only one subset of 50 environments within the choice for *V_env_* =70 was used for sampling an ensemble with *V_env_* =50. However, for *V_env_* =40, we have taken 10 different subsets of 40 environments within the set used for *V_env_* =50; thus we generated samples in 10 different ensembles at *V_env_* =40. (Note that for clarity we have chosen to display only two different subsets for *V_env_* =40 in this figure). For *V_env_* =30, we used 10 subsets of 30 environments, one chosen from each of the sets used at *V_env_* =40, thereby sampling 10 different ensembles at *V_env_* =30. Similarly, for *V_env_* =20, we took 10 subsets of 20 environments, one from each of the 10 sets at *V_env_* =30, producing 10 different ensembles with *V_env_* =20. The same procedure was followed for *V_env_* =10, 5, 2 and 1. In summary, we took 10 different choices of nested environments to sample genotypes in the ensembles with *V_env_* = 1, 2, 5, 10, 20, 30 and 40, and averaged the properties of the sampled genotypes over the 10 different choices for these *V_env_* values. Note that the higher the phenotypic constraints are (the more environments we tested), the longer the computation times become for sampling genotypes in the ensemble of interest. That is why we have used just one set of environments for *V_env_* =50 and 70.
